# Image compression-encryption method based on two-dimensional sparse recovery and chaotic system

**DOI:** 10.1038/s41598-020-79747-4

**Published:** 2021-01-11

**Authors:** Aboozar Ghaffari

**Affiliations:** Department of Electrical Engineering, Iran University of Science and Technology, Tehran, Iran

**Keywords:** Biomedical engineering, Electrical and electronic engineering

## Abstract

In this paper, we propose an image compression-encryption method based on two-dimensional (2D) sparse representation and chaotic system. In the first step of this method, the input image is extended in a transform domain to obtain a sparse representation. To achieve better performance of image compression by 2D sparse recovery, the sparse representation is scrambled via a chaotic confusion. This step helps the satisfaction of the uniqueness conditions for sparse recovery, and the security level of encryption is increased. Then, two orthogonal measurement matrices are generated using the chaotic time series. The singular value decomposition is used to compress the sparse scrambled representation in two dimensions. Finally, to reduce the correlation between adjacent pixels in the compressed matrix, and obtain a uniform distribution in the encrypted image, a compressed scrambling matrix based on chaotic confusion is used. Then, XOR operation is applied for final encryption. In the decryption process, to improve the compression efficiency, the total variation constraint is added to the 2D sparse recovery problem based on the smoothed norm. The simulation results demonstrate the satisfying performance of the proposed method for different compression ratios. Security analysis describes the effectiveness of the proposed encryption approach.

## Introduction

Recently, by the development of technology and network, images are widely used in various applications. Since the images are usually needed to transmit in an insecure channel such as the internet, protection of images from various attacks by hackers is an essential task in various applications such as health care^1,2^, multimedia systems, and military^3^. Image encryption is an important approach to convert data to a meaningless form that its understanding is difficult^3,4^. Image encryption approaches are categorized into symmetric and asymmetric key cryptography^5,6^. Recently, different architectures were proposed to encryption images such as optical image encryption^7,8^, DNA coding^9,10^, XOR operation^11,12^ and quantum image encryption^13–16^. Since chaotic systems are sensitive to the initial conditions, various encryption algorithms based on chaotic systems were proposed. So, their initial conditions are used as a key in the encryption process^13,17–20^. Usually, the encrypted image is like a noisy image or texture image. Recently, the visually meaningful encryption schemes were proposed that have used a carrier image to encrypt and transmit the original image^21,22^.

In the transmission network, the original image should be encrypted and compressed due to the limited bandwidth and illegal eavesdropping. Recently, the image encryption based on compressed sensing (CS) or sparse representation has been a hot topic^23–26^. These approaches have assumed that the original image has a sparse representation in a transform domain such as wavelet or discrete cosine transform (DCT). Then the sparse representation is compressed by a measurement matrix, which is a part of the encryption key. In the decryption process, the original image is recovered by an optimization problem of sparse recovery. To obtain better security performance, sparse representation and traditional approaches were combined. Visually meaningful encryption schemes^22,27,28^, chaotic map^29–36^, cellular automata^37,38^, and discrete fractional random transform^39^ are some examples.

An essential measure of CS-based encryption schemes is compression and reconstruction performance. Hence, many works have been done to obtain better reconstruction. In sparse recovery, the transform which is used to obtain sparse representation has a critical role. Many different transforms, such as wavelet^34,40^, DCT^38^, and learned dictionary^35^, were used in different approaches. The transforms also can be categorized into block-based transform^35^ and global-based transform^21,34^. In Ref.^41^, it was shown that the scrambling of sparse coefficients could improve compression and reconstruction performance. Some approaches have used the zigzag scan for random permutation of the sparse coefficients^38,41,42^. To reduce the computational complexity and required memory, in the sparse recovery literature, sparse decomposition of the two-dimensional signal^43^ or Kronecker compressed sensing^44^ based on two measurement matrices in two directions has been proposed. Hence, some proposed encryption approaches have used two measurement matrices to obtain better compression and reconstruction performance^34,39,45^. Also, designing the measurement matrix is a modification approach to obtain better performance. Usually, CS-based encryption approaches use chaotic systems to generate this matrix. Some approaches construct the measurement matrix based on the combination of the Hadamard transform and chaotic map^22,38,39,45^.

In this paper, an image encryption approach based 2D sparse recovery is proposed. In Ref.^43^, the uniqueness conditions of 2D sparse recovery were investigated based on the spark definition. The main properties of these conditions are that the uniformity of sparse coefficients in rows and columns improves the uniqueness conditions to obtain better compression and sparse reconstruction. Based on this theory, a random permutation using a chaotic Lorenz system is proposed. Random orthogonal measurement matrices are constructed via the Lorenz system and singular value decomposition. Simulation results show that the proposed method has an acceptable performance. In this algorithm, the total variation is proposed in the sparse recovery problem based on smoothed $\ell_{0}$ norm SL0^43,46^. In the decryption process, a sparse recovery approach based on the SL0, total variation, and scrambling of sparse coefficients is proposed. This approach improves the compression and reconstruction performance effectively in comparison with other approaches at the same compression ratio. The results show that the proposed approach is robust in the presence of a cropping attack. To obtain the meaningless image in the encryption process, XOR operation and the Lorenz system are used. Security analyses illustrate the effectiveness of the proposed encryption approach. In the section of preliminaries, the concept of 2D sparse recovery and Lorenz system are described. The main idea and the proposed method are stated in the section of the proposed encryption method. The section of experimental results provides security analyses. Finally, the paper is concluded, and the future work direction is pointed out.

## Preliminaries

### Sparse representation

An important core of signal processing is signal decomposition representing a signal vector ${\mathbf{y}} \in R^{n}$ using a linear combination of some basis functions $\phi_{i} , 1 \le i \le m$, that is ${\mathbf{y}} = s_{1} \phi_{1} + \ldots + s_{m} \phi_{{\text{m}}} = {\mathbf{\Phi s}}$, where ${{\varvec{\Phi}}} = \left[ {{{\varvec{\Phi}}}_{1} , \ldots ,{{\varvec{\Phi}}}_{m} } \right]$, and ${\mathbf{s}} = \left[ {s_{1} , \ldots ,s_{m} } \right]^{T}$. Recently, the sparsity assumption on signal and image representation (${\mathbf{s}}$) has been used for various applications such as image denoising^47^, blind source separation^48^, compressed sensing^24^, pattern recognition^49^, and image registration^50,51^. It is assumed that the signal is k-sparse, which means it has at most k non-zero entries in a learned dictionary or a transform domain such as discrete cosine transform or wavelet.

Sparsity assumption is the basis of compressed sensing. It has a crucial role in our proposed method of image encryption. The goal of compressed sensing is to recover an unknown sparse signal ${\mathbf{s}} \in R^{m}$ from a set of underdetermined measurements ${\mathbf{y}} = {\mathbf{\Phi s}} \in R^{n} (m < n)$ where ${{\varvec{\Phi}}} \in {\text{R}}^{n \times m}$ is the measurement matrix. Sparse recovery problem can be formulated as:

1 $$P_{0}^{1D} : {\text{Minimize}} |\left| {\mathbf{s}} \right||_{0} s.t \,\,\,\,{\mathbf{y}} = {\mathbf{\Phi s}}$$

where $|\left| {\mathbf{s}} \right||_{0}$ stands for the $\ell_{0}$ norm of **s** that is the number of non-zero elements of **s**. In this problem, there are two fundamental questions as follows:

1. Uniqueness: does the problem $P_{0}^{1D}$ have a unique solution?
2. Practical solver: does the optimization problem of the sparsest solution have a practical solver?

In reply to the first question, it has been shown that under some conditions, the sparsest solution is unique. Here, we state the uniqueness theorem based on the spark definition.

Theorem (Uniqueness^52^): Let $spark\left( {{\varvec{\Phi}}} \right)$ denote the minimum number of columns of ${{\varvec{\Phi}}}$ that are linearly dependent. Then if the equation ${\mathbf{y}} = {\mathbf{\Phi s}}$ has a solution ${\mathbf{s}}_{0}$ for which ${\mathbf{s}}_{0} < spark\left( {{\varvec{\Phi}}} \right)/2$, it is its unique sparsest solution.

Although the sparsest solution of (1) may be unique, finding it requires a combinatorial search and is generally NP-hard. Several methods have been developed in recent years such as matching pursuit^53^, basis pursuit^54^ and smoothed $\ell_{0}$ (SL0)^46^.

In some application, we need to use compressive sensing on two dimensional (2D) signal. To achieve this goal, a trivial approach is to convert the 2D problem to the above one-dimensional (1D) problem. Two disadvantages of this approach are the computational load and the high required memory. To overcome these problems, two measurement matrices ${{\varvec{\Phi}}}_{1} \in R^{{n_{1} \times m_{1} }}$ and ${{\varvec{\Phi}}}_{2} \in R^{{n_{2} \times m_{2} }}$ were used to compress a sparse matrix ${\mathbf{S}} \in R^{{m_{1} \times m_{2} }}$ in the form of ${\mathbf{Y}} = {{\varvec{\Phi}}}_{1} {\mathbf{S\Phi }}_{2}^{{\mathbf{T}}} \in R^{{n_{1} \times n_{2} }}$^43^. The sparse recovery based on two measurement matrices can be obtained by solving the following optimization problem.

2 $$P_{0}^{2D} : {\text{Minimize}} |\left| {\mathbf{S}} \right||_{0} s.t {\mathbf{Y}} = {{\varvec{\Phi}}}_{1} {\mathbf{S\Phi }}_{2}^{{\mathbf{T}}}$$

The uniqueness of this problem has been shown in some researches^43,55,56^. In Ref.^43^, the uniqueness conditions of this sparse recovery problem based on spark definition were investigated. It has been shown that the necessary (but not sufficient) conditions about the distribution of non-zero entries of **S** are as follows:

- $$\left\| {{\mathbf{S}}_{0} } \right\| < \frac{{spark\left( {{{\varvec{\Phi}}}_{1} } \right)spark\left( {{{\varvec{\Phi}}}_{2} } \right)}}{4}$$
- ($\ell_{0}$ norm of each column)$< \frac{{spark\left( {{{\varvec{\Phi}}}_{1} } \right)}}{2}$
- ($\ell_{0}$ norm of each row)$< \frac{{spark\left( {{{\varvec{\Phi}}}_{2} } \right)}}{2}$

Some sparse recovery algorithms have been proposed to solve 2D compressed sensing such as 2D orthogonal matching pursuits (2D-OMP)^57^, 2D projected gradient (2DPG) algorithm^58^, and 2D smoothed $\ell_{0}$ (2D-SL0)^43^.

In this paper, a new approach of image encryption based on 2D smoothed $\ell_{0}$ norm is proposed. The idea of SL0 is to replace $\ell_{0}$ norm with a smooth approximation of $\ell_{0}$ norm such as ${\mathbf{S}}_{0} \approx {\mathbf{S}}_{\sigma 0} = \sum\nolimits_{i,j} {\left( {1 - \exp \left( { - \frac{{s_{ij}^{2} }}{{2\sigma^{2} }}} \right) } \right)}$. 2DSL0 tries to solve directly $P_{0}^{2D}$ problem. The smoothed $\ell_{0}$ norm is equivalent to $\ell_{0}$ norm when $\sigma \to 0$. 2DSL0 minimizes ${\mathbf{S}}_{\sigma 0}$ for a small $\sigma$, subject to ${\mathbf{Y}} = {{\varvec{\Phi}}}_{1} {\mathbf{S\Phi }}_{2}^{{\mathbf{T}}}$. In this paper, to improve the performance of sparse recovery, the 2DSL0 problem is regularized with the total variation (TV).

### Lorenz system

**Figure 1:**
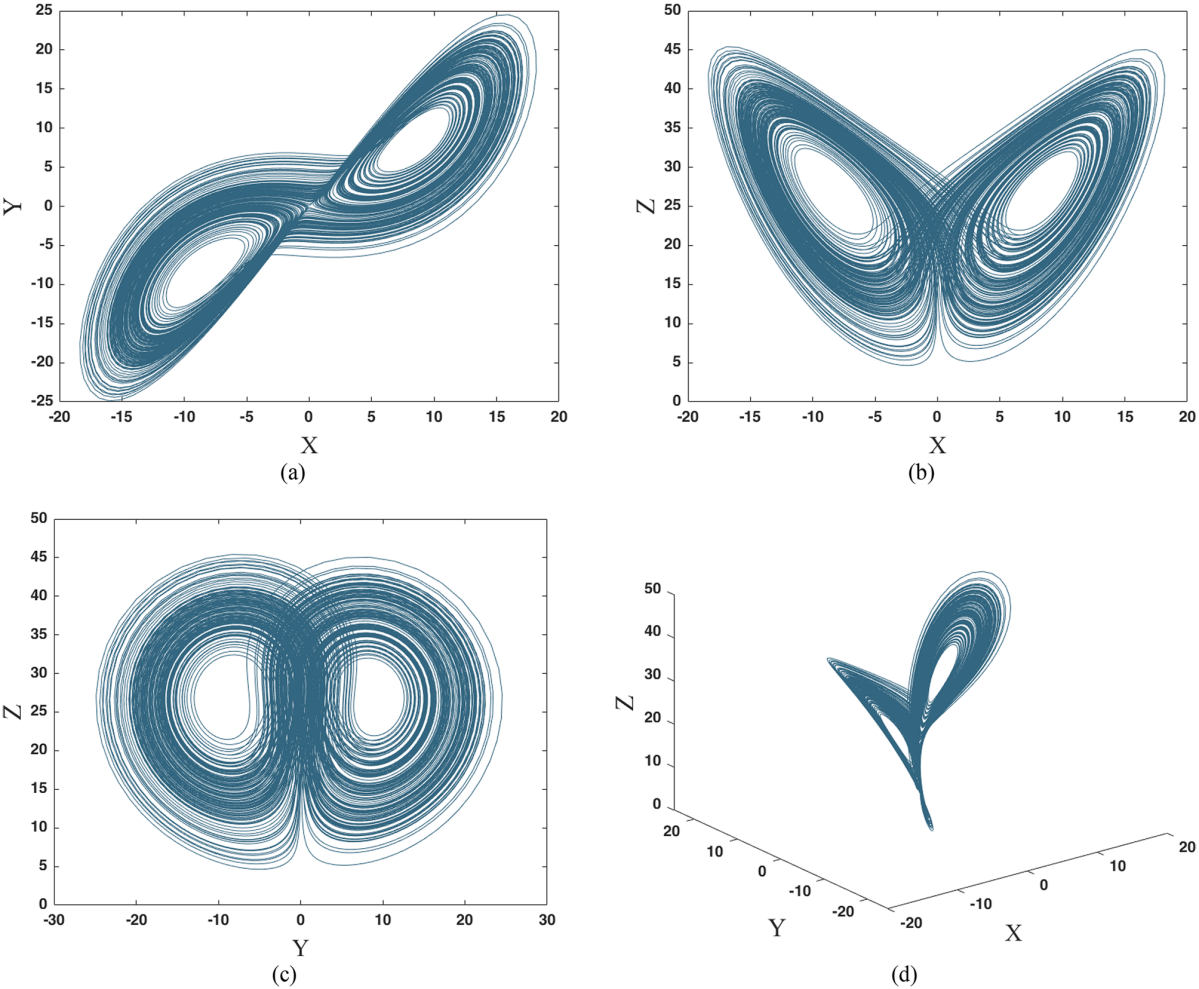
Chaotic attractor of Lorenz system in parameters $\sigma = 10$, $\beta = \frac{8}{3}$ and $\rho = 28$, and initial conditions $\left[ {x_{01} ,y_{01} ,z_{01} \left] = \right[ - 0.823 ,3.423 , 0.178} \right]$; (**a**) 2D projection of chaotic attractor in $X - Y$ plane; (**b**) 2D projection of chaotic attractor in $X - Z$ plane; (**c**) 2D projection of chaotic attractor in $Y - Z$ plane; (**d**) 3D chaotic attractor; these attractors are generated by ODE15s in MATLAB 2018a.

Lorenz system is one of the well-known chaotic systems which is highly sensitive to initial conditions^59^. It means that a small change in initial values can cause a considerable change in the final state of the time series. The Lorenz system is presented in Eq. (3). The chaotic attractor of the Lorenz system in parameters $\sigma = 10$, $\beta = \frac{8}{3}$ and $\rho = 28$, and initial conditions $\left[ {x_{01} ,y_{01} ,z_{01} \left] = \right[ - 0.823 ,3.423 , 0.178} \right]$ is shown in Fig. 1.

3 $$\begin{array}{*{20}c} {\dot{x} = \sigma \left( {y - x} \right)} \\ {\dot{y} = x\left( {\rho - z} \right) - y} \\ {\dot{z} = xy - \beta z} \\ \end{array}$$

### The proposed encryption method

**Figure 2:**
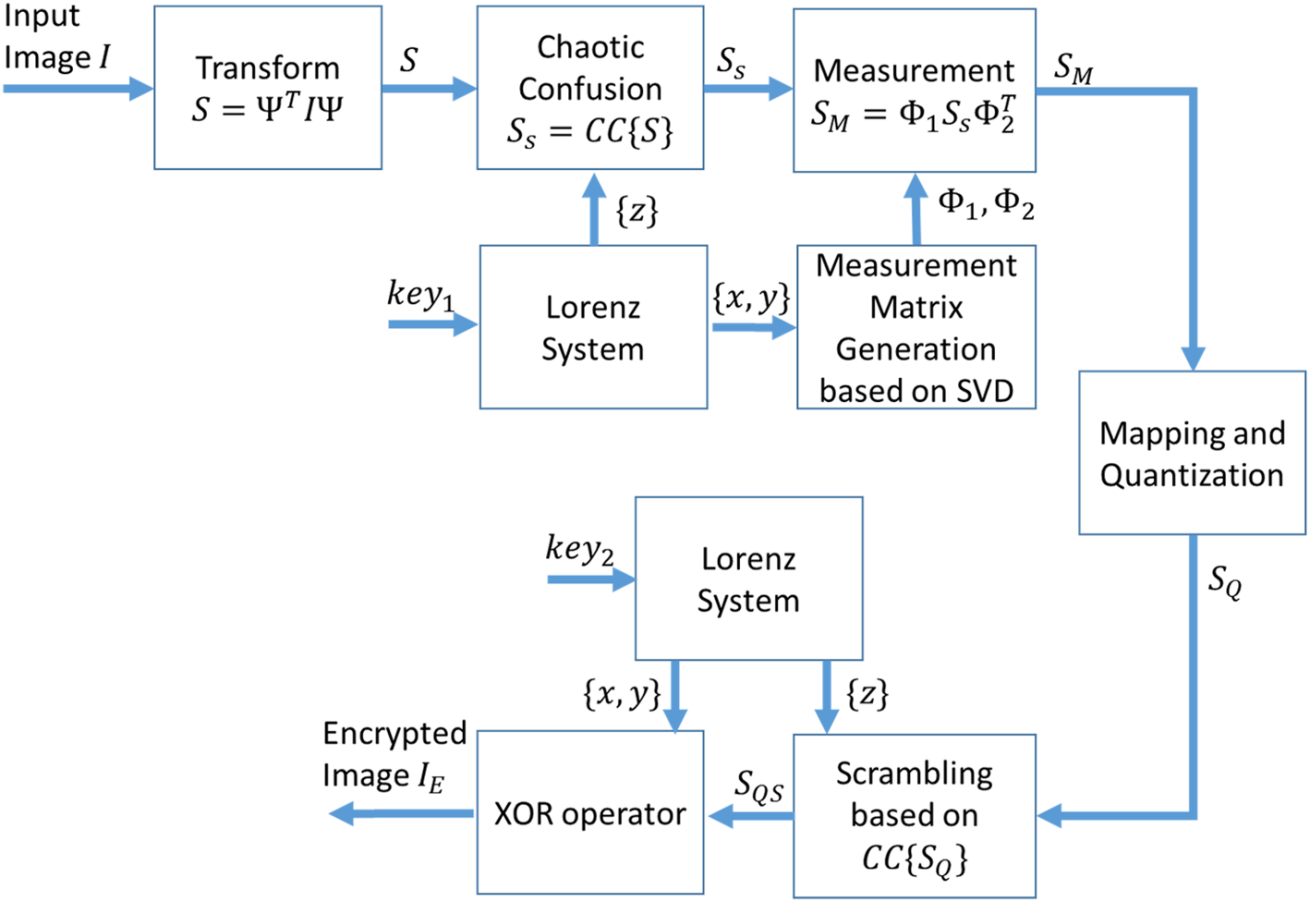
The block diagram of the proposed encryption approach.

In this section, we propose a new image encryption algorithm based on two-dimensional compressed sensing and the chaotic Lorenz system. The proposed algorithm consists of two steps: compression via 2D compressed sensing and scrambling based on pixel shuffling and XOR operator. Figure 2 illustrates the block diagram of the image compression-encryption algorithm. In the rest of this section, at first two blocks of confusion based on chaotic and constructing measurement matrix are demonstrated, then the encryption process is described.

### Chaotic confusion

In the previous section, we investigate the uniqueness conditions of 2D sparse representation based on the distribution of non-zero entries of the sparse representation matrix. To achieve these conditions, the sparse coefficient matrix is confused by shuffling based on the chaotic system. This confusion also decreases the correlation among elements of the sparse matrix to increase the security level of image encryption. The steps of chaotic confusion (CC) are described as follows:

1. Generating the sequence $X = \left\{ {{\varvec{x}}_{{\varvec{i}}} } \right\}$ of the Lorenz system for an initial condition with a suitable step size in the Runge–Kutta method. The length of $X$ is equal to the number of sparse matrix elements.
2. Transforming the sequence $X = \left\{ {{\varvec{x}}_{{\varvec{i}}} } \right\}$ into integer sequence $X^{*} = \left\{ {{\varvec{x}}_{{\varvec{i}}}^{*} } \right\}$:
  $$x_{i}^{*} = mod\left( {x_{i} \times 10^{15} ,2^{16} } \right)$$
3. Sorting the sequence ${\text{X}}^{*}$:
  $$\left[ {X_{s}^{*} ,idx_{s}^{X} } \right] = sort\left( {X^{*} } \right)$$
  where [. , .] = sort(.) is the sequencing operator, $X_{s}^{*}$ is the sorted sequence. $idx_{s}^{X}$ is the index sequence of $X_{s}^{*}$ containing a sequence of integer numbers with a random perturbation.
4. Shuffling the entries of the sparse matrix according to the sequence $idx_{s}^{X}$.

We also use this approach in the scrambling step of the image encryption algorithm.

### Chaotic measurement matrix generation

The measurement matrix has a crucial task in compressed sensing. In the proposed method, at first, the original image is transformed into a sparse domain via DWT. Then the sparse matrix ${\mathbf{S}} \in R^{{n_{1} \times n_{2} }}$ is measured and compressed using two measurement matrices ${{\varvec{\Phi}}}_{1} \in R^{{{\text{m}}_{1} \times n_{1} }}$ and ${{\varvec{\Phi}}}_{2} \in R^{{m_{2} \times n_{2} }}$. Here, to generate the measurement matrices, we use a unitary matrix constructed by a random matrix based on the chaotic system. The state variables $Y = \left\{ {{\varvec{y}}_{{\varvec{i}}} } \right\}$ and $Z = \left\{ {{\varvec{z}}_{{\varvec{i}}} } \right\}$ of the Lorenz system are used to generate measurement matrices ${{\varvec{\Phi}}}_{1}$ and ${{\varvec{\Phi}}}_{2}$, respectively. The generation process of the two matrices is similar. Five steps of matrix generation for ${{\varvec{\Phi}}}_{1}$ are describes as follows:

1. Generating the sequence $Y = \left\{ {{\varvec{y}}_{{\varvec{i}}} } \right\}$ of the Lorenz system for an initial condition with a suitable step length in the Runge–Kutta method. The length of $Y$ is equal to $n_{1}^{2}$.
2. Transforming the sequence $Y = \left\{ {{\varvec{y}}_{{\varvec{i}}} } \right\}$ into integer sequence $Y^{*} = \left\{ {{\text{y}}_{{\text{i}}}^{*} } \right\}$:
  $$y_{i}^{*} = mod\left( {y_{i} \times 10^{15} ,2^{16} } \right)$$
3. Reshaping the sequence $Y^{*}$ to construct the matrix ${\mathbf{Y}}_{{\mathbf{M}}} \in R^{{n_{1} \times n_{1} }}$ which is a random matrix generated by a chaotic system.
4. Computing the singular value decomposition:
  $${\mathbf{Y}}_{{\mathbf{M}}} = {\mathbf{U\Sigma V}}^{{\mathbf{T}}}$$
  where ${{\varvec{\Sigma}}} \in R^{{n_{1} \times n_{1} }}$ is a diagonal matrix containing singular values. The matrices ${\mathbf{U}} \in R^{{n_{1} \times n_{1} }}$ and ${\mathbf{V}} \in R^{{n_{1} \times n_{1} }}$ are unitary in which their columns are singular vectors.
5. Constructing the measurement matrix ${{\varvec{\Phi}}}_{1}$ by selecting the first $m_{1}$ rows of matrix ${\mathbf{U}}$.

### Image encryption and compression based on 2D sparse representation

Here, we investigate the proposed encryption process (Fig. 2) as follows:

**Step 1:** The original image ${\mathbf{I}} \in R^{{n_{1} \times n_{2} }}$ is extended in a transform domain ${{\varvec{\Psi}}}$ such as DWT, to obtain the sparse representation ${\mathbf{S}}$:

$${\mathbf{S}} = {{\varvec{\Psi}}}^{{\mathbf{T}}} {\mathbf{I\Psi }} \in R^{{n_{1} \times n_{2} }}$$

**Step 2:** The chaotic confusion is used to scramble the sparse matrix ${\mathbf{S}}$:

$${\mathbf{S}}_{{\mathbf{s}}} = CC\left\{ {\mathbf{S}} \right\}$$

where $CC\left\{ . \right\}$ is a chaotic confusion operator.

**Step 3:** The sparse matrix ${\mathbf{S}}_{{\mathbf{s}}}$ is measured using two chaotic measurement matrices ${{\varvec{\Phi}}}_{1} \in R^{{m_{1} \times n_{1} }}$ and ${{\varvec{\Phi}}}_{2} \in R^{{m_{2} \times n_{2} }}$:

$${\mathbf{S}}_{{\mathbf{M}}} = {{\varvec{\Phi}}}_{1} {\mathbf{S}}_{{\mathbf{s}}} {{\varvec{\Phi}}}_{2}^{{\mathbf{T}}} \in R^{{m_{1} \times m_{2} }}$$

In our simulation, it is assumed that the compression ratio is the same in two dimensions, i.e. $\frac{{m_{1} }}{{n_{1} }} = \frac{{m_{2} }}{{n_{2} }}$. Hence total compression ratio (CR) is equal to the square of the CR in each dimension.

**Step 4**: The measured matrix ${\mathbf{S}}_{{\mathbf{M}}}$ is mapped and quantized in the range of [0,255] using the following formula:

$${\mathbf{S}}_{{\mathbf{Q}}} = \left[ {\frac{{255({\mathbf{S}}_{{\mathbf{M}}} - {\text{min}}\left( {{\mathbf{S}}_{{\mathbf{M}}} } \right)}}{{\max \left( {{\mathbf{S}}_{{\mathbf{M}}} } \right) - \min \left( {{\mathbf{S}}_{{\mathbf{M}}} } \right)}}} \right]$$

where $\left[ x \right]$ rounds the entry of $x$ to the nearest integer value. In this mapping, each pixel of ${\mathbf{S}}_{{\mathbf{Q}}}$ can be represented by one byte.

**Step 5:** The chaotic confusion is used to scramble the quantized matrix $S_{Q}$:

$${\mathbf{S}}_{{{\mathbf{QS}}}} = CC\left\{ {{\mathbf{S}}_{{\mathbf{Q}}} } \right\}$$

This step increases security level by reducing the correlation among the elements of the quantized matrix ${\mathbf{S}}_{{\mathbf{Q}}}$.

**Step 6**: In the final step, to obtain a uniform distribution for the encrypted image, the scrambled matrix ${\mathbf{S}}_{{{\mathbf{QS}}}}$ is XORed with a random sequence generated by the Lorenz system. This step is investigated as follows:

- Generating the sequences $Y = \left\{ {{\varvec{y}}_{{\varvec{i}}} } \right\}$ and $Z = \left\{ {{\varvec{z}}_{{\varvec{i}}} } \right\}$ of Lorenz system for an initial condition with a suitable step length in the Runge–Kutta method. The length of $Y$ and $Z$ is equal to $m_{1} m_{2}$.
- Two sequences $Y$ and $Z$ are transformed into integer number in the range of [0,255]:
  $$y_{i}^{*} = mod\left( {y_{i} \times 10^{15} ,2^{8} } \right)$$
  $$z_{i}^{*} = mod\left( {z_{i} \times 10^{15} ,2^{8} } \right)$$
- The matrix ${\mathbf{S}}_{{{\mathbf{QS}}}}$ and sequences $Y^{*} = \left\{ {y_{i}^{*} } \right\}$ and $Z^{*} = \left\{ {z_{i}^{*} } \right\}$ are converted to the binary with $8$ bits. Then the encrypted image is obtained by XOR operator:
  $${\mathbf{I}}_{{\mathbf{E}}} = {\mathbf{S}}_{{{\mathbf{QS}}}} \oplus \left[ {Y^{*} \oplus Z^{*} } \right]$$

Finally, the image is converted to the decimal.

In the proposed image encryption, the Lorenz system with two different sets of initial conditions is used to generate six chaotic sequences.

### The decryption process and sparse recovery

The decryption process is the inverse operator of the encryption to recover the original image. Here, we assume that the receiver has the initial conditions and step size of the chaotic system to generate random sequences required for the construction of the measurement matrix and the scrambling operators. This process is described as follows:

**Step 1**: Obtaining ${\mathbf{S}}_{{{\mathbf{QS}}}}$ from ${\mathbf{I}}_{{\mathbf{E}}}$ by the XOR operator:

$${\mathbf{S}}_{{{\mathbf{QS}}}} = {\mathbf{I}}_{{\mathbf{E}}} \oplus \left[ {Y^{*} \oplus Z^{*} } \right]$$

**Step 2**: After the inverse chaotic confusion of image $S_{QS}$, the measured matrix ${\mathbf{S}}_{{\mathbf{M}}}$ is obtained by the inverse mapping as follows:

$${\mathbf{S}}_{{\mathbf{M}}} = \frac{{CC^{ - 1} \{ {\mathbf{S}}_{{{\mathbf{QS}}}} \} \left( {\max \left( {{\mathbf{S}}_{{\mathbf{M}}} } \right) - \min \left( {{\mathbf{S}}_{{\mathbf{M}}} } \right)} \right)}}{255} + {\text{min}}\left( {{\mathbf{S}}_{{\mathbf{M}}} } \right)$$

where $CC^{ - 1} \{_{.} \}$ is the inverse operator of chaotic confusion. $\max \left( {{\mathbf{S}}_{{\mathbf{M}}} } \right)$ and $\min \left( {{\mathbf{S}}_{{\mathbf{M}}} } \right)$ are also gotten from the sender.

**Step 3**: The sparse matrix ${\mathbf{S}}_{{\mathbf{s}}}$ is obtained from the measured matrix ${\mathbf{S}}_{{\mathbf{M}}}$ using a sparse reconstruction algorithm based on the 2DSL0 algorithm. In this paper, we improve the performance of sparse recovery by adding total variation (TV) regularization term to the cost function of 2DSL0. This step is investigated in the next subsection.

**Step 4**: After the inverse chaotic confusion of the sparse matrix ${\mathbf{S}}_{{\mathbf{S}}}$, the original image ${\mathbf{I}}$ is obtained by the inverse transform ${{\varvec{\Psi}}}$ (IDWT), and the decryption process is finished.

### Sparse recovery based on 2D smoothed $\ell_{0}$ norm and total variation

In this subsection, we propose 2D sparse recovery based on smoothed $\ell_{0}$ norm and total variation. We know that the natural images are usually smooth, hence we add this information to the 2D sparse recovery problem via total variation. To obtain the better performance of sparse reconstruction, the search space of the sparse optimization problem is limited by adding the TV regularization to the cost function of sparse measure. Here, the following definition of TV is used to measure the smoothness of the estimated original image ${\hat{\mathbf{I}}}$:

4 $$TV\left( {{\hat{\mathbf{I}}}} \right) = \mathop \sum \limits_{i,j} \left| {\frac{{\partial {\hat{\mathbf{I}}}}}{\partial x}\left( {i,j} \right)} \right| + \left| {\frac{{\partial {\hat{\mathbf{I}}}}}{\partial y}\left( {i,j} \right)} \right|$$

where the image ${\hat{\mathbf{I}}}$ is the inverse transform of the estimated sparse matrix ${\hat{\mathbf{S}}}$, i.e. ${\hat{\mathbf{I}}} = {\mathbf{\Psi}} {{\hat{\mathbf{S}}}{\mathbf{\Psi }}^{{\mathbf{T}}}}$. The absolute function is not differentiable at the origin; hence we replace the absolute function of $x$ with the smoothed function $\sqrt {x^{2} + \delta }$ where $\delta$ is a small value such as 0.001. The derivative is estimated discretely via forwarding difference. Let the square matrix ${\mathbf{D}}$ be in the following form:

5 $${\mathbf{D}} = \left[ {\begin{array}{*{20}c} {\begin{array}{*{20}c} { - 1} \\ 0 \\ {\begin{array}{*{20}c} 0 \\ {\begin{array}{*{20}c} 0 \\ 0 \\ \end{array} } \\ \end{array} } \\ \end{array} } & {\begin{array}{*{20}c} {\begin{array}{*{20}c} 1 \\ { - 1} \\ {\begin{array}{*{20}c} 0 \\ 0 \\ 0 \\ \end{array} } \\ \end{array} } & {\begin{array}{*{20}c} {\begin{array}{*{20}c} 0 \\ 1 \\ {\begin{array}{*{20}c} \ddots \\ 0 \\ 0 \\ \end{array} } \\ \end{array} } & {\begin{array}{*{20}c} {\begin{array}{*{20}c} 0 \\ 0 \\ {\begin{array}{*{20}c} \ddots \\ { - 1} \\ 0 \\ \end{array} } \\ \end{array} } & {\begin{array}{*{20}c} 0 \\ 0 \\ {\begin{array}{*{20}c} 0 \\ 1 \\ 0 \\ \end{array} } \\ \end{array} } \\ \end{array} } \\ \end{array} } \\ \end{array} } \\ \end{array} } \right]$$

The derivative of the estimated original image ${\hat{\mathbf{I}}}$ can be written in the following matrix form:

6 $$\begin{gathered} \frac{{\partial {\hat{\mathbf{I}}}}}{\partial x} = {\mathbf{D}}_{{\mathbf{x}}} {\hat{\mathbf{I}}} = {\mathbf{D}}_{{\mathbf{x}}} {\mathbf{\Psi}} {{\hat{\mathbf{S}}}{\mathbf{\Psi }}^{\mathbf{T}}} \hfill \\ \frac{{\partial {\hat{\mathbf{I}}}}}{\partial y} = {\hat{\mathbf{I}}\mathbf{D}}_{{\mathbf{y}}}^{{\mathbf{T}}} = {\mathbf{\Psi}} {{\hat{\mathbf{S}}}{\mathbf{\Psi }}^{\mathbf{T}}} {\mathbf{D}}_{{\mathbf{y}}}^{{\mathbf{T}}} \hfill \\ \end{gathered}$$

where ${\mathbf{D}}_{{\mathbf{x}}} \in R^{{n_{1} \times n_{1} }}$ and ${\mathbf{D}}_{{\mathbf{y}}} \in R^{{n_{2} \times n_{2} }}$ have the form of matrix ${\mathbf{D}}$. Based on the above definition, the regularized 2D sparse recovery using SL0 is proposed in the following form:

7 $$\arg \mathop {\min }\limits_{{S_{s} }} J_{\sigma } \left( {{\mathbf{S}}_{{\mathbf{s}}} } \right) = \mathop \sum \limits_{i,j} \left( {1 - \exp \left( { - \frac{{s_{ij}^{2} }}{{2\sigma^{2} }}} \right) } \right) + \lambda TV\left( {\mathbf{I}} \right), \text{subject to } \quad {\mathbf{S}}_{{\mathbf{M}}} = {{\varvec{\Phi}}}_{1} {\mathbf{S}}_{{\mathbf{s}}} {{\varvec{\Phi}}}_{2}^{{\mathbf{T}}} , {\mathbf{I}} = {{\varvec{\Psi}}} \times {\mathbf{CC}}^{ - 1} \left\{ {{\mathbf{S}}_{{\mathbf{s}}} } \right\} \times {{\varvec{\Psi}}}^{{\mathbf{T}}}$$

where $\lambda > 0$ is the Lagrange coefficient to quantify the trade-off between sparsity in the transform domain and TV. In this problem, to recover the original image, two sparsity constraints are used in two different domains of the transform ${ }{{\varvec{\Psi}}}$ and the image gradient. In this optimization, the final two steps of image decryption (Steps 3 and 4) are combined with the TV regularization. Inspired by Refs.^43,46^, to escape from trapping into local minima, the sparsity measure is optimized iteratively by using a decreasing sequence of $\sigma$. The minimizer of $J_{\sigma } \left( {{\mathbf{S}}_{{\mathbf{s}}} } \right)$ is used as a starting point to minimize $J_{\sigma } \left( {{\mathbf{S}}_{{\mathbf{s}}} } \right)$ for the smaller $\sigma$. To optimize $J_{\sigma } \left( {{\mathbf{S}}_{{\mathbf{s}}} } \right)$ for a fixed $\sigma$ subject to the condition ${\mathbf{S}}_{{\mathbf{M}}} = {{\varvec{\Phi}}}_{1} {\mathbf{S}}_{{\mathbf{s}}} {{\varvec{\Phi}}}_{2}^{{\mathbf{T}}}$, the optimizer is based on the steepest descent approach in which each of its iteration $\left( {{\mathbf{S}}_{{\mathbf{s}}} \leftarrow {\mathbf{S}}_{{\mathbf{s}}} - \mu \nabla_{{{\text{S}}_{{\text{s}}} }} {\text{J}}_{{\upsigma }} \left( {{\mathbf{S}}_{{\mathbf{s}}} } \right)} \right)$ followed by projection onto the feasible set $\left\{ {{\mathbf{S}}_{{\mathbf{s}}} |{\mathbf{S}}_{{\mathbf{M}}} = {{\varvec{\Phi}}}_{1} {\mathbf{S}}_{{\mathbf{s}}} {{\varvec{\Phi}}}_{2}^{{\mathbf{T}}} } \right\}$. The more details about this optimization approach can be found in the literature^43,46^. Required gradient of the TV for this optimization can be computed with simple calculations as follows:

8 $$\begin{gathered} \frac{{\partial \left( {\frac{{\partial {\mathbf{I}}}}{\partial x}} \right)}}{{\partial S_{s} }} = CC\left\{ {{{\varvec{\Psi}}}^{{\varvec{T}}} {\varvec{D}}_{{\varvec{x}}}^{{\varvec{T}}} \hat{\user2{S}}{{\varvec{\Psi}}}} \right\} \hfill \\ \frac{{\partial \left( {\frac{{\partial {\mathbf{I}}}}{\partial y}} \right)}}{{\partial S_{s} }} = CC\left\{ {{{\varvec{\Psi}}}^{{\mathbf{T}}} {\hat{\mathbf{S}}\mathbf{D}}_{{\mathbf{y}}} {{\varvec{\Psi}}}} \right\} \hfill \\ {\hat{\mathbf{S}}} = CC^{ - 1} \left\{ {\frac{{{\mathbf{S}}_{{\mathbf{s}}} }}{{\sqrt {{\mathbf{S}}_{s}^{2} + \delta } }}} \right\} \hfill \\ \end{gathered}$$

To consider the noise effect in the proposed sparse recovery problem, the equality constraint ${\mathbf{S}}_{{\mathbf{M}}} = {{\varvec{\Phi}}}_{1} {\mathbf{S}}_{{\mathbf{s}}} {{\varvec{\Phi}}}_{2}^{{\mathbf{T}}}$ is used in the relaxed form ${\mathbf{S}}_{{\mathbf{M}}} - {\mathbf{\Phi }}_{1} {\mathbf{S}}_{{\mathbf{s}}} {\mathbf{\Phi }}_{{2F}}^{{\mathbf{T}}} \le \epsilon$. Inspired by Ref.^60^, this condition is considered by performing the projection process when the constraint ${\mathbf{S}}_{{\mathbf{M}}} - {\mathbf{\Phi }}_{1} {\mathbf{S}}_{{\mathbf{s}}} {\mathbf{\Phi }}_{{2F}}^{{\mathbf{T}}} \le \epsilon$ is not satisfied. The parameter $\epsilon$ is set dependent on the noise level. The image decryption approach based of 2DSL0 and total variation is illustrated in Algorithm 1. The parameters for the proposed approach are empirically selected as fallows: $\lambda = 0.01$, $L = 5$, $\mu = 0.5$, $\delta = 0.001$, $\epsilon = 0.001$. The parameter $\sigma = \left[ {\sigma_{1} , \ldots ,\sigma_{J} } \right]$ is selected as $\sigma_{1} = \max \left( {\left| {{\hat{\mathbf{S}}}_{{{\mathbf{s}}0}} } \right|} \right)$, and $\sigma_{j} = \sigma_{1} \left( {1 - 0.003j} \right)$ which is a geometric decreasing sequence.
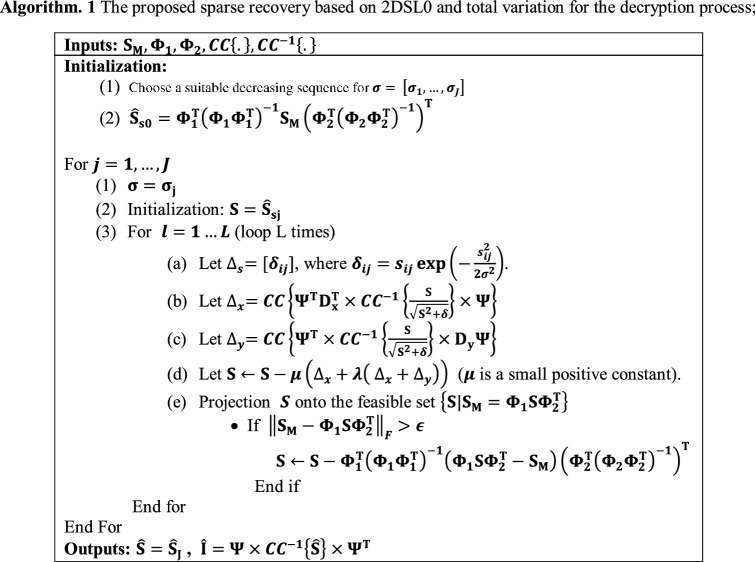


## Experimental results

In this section, the proposed method is evaluated by different measures. All the experiments are performed on a personal computer with Intel Core i7 2.2 GHz and 8 GB RAM. The images of Cameraman, Pepper, Baboon and Brain with a size of $256 \times 256$ are used for evaluation. The DWT is used as a sparsifying transform. In the simulations, the parameters of the Lorenz system are constant as $\sigma = 10$, $\beta = \frac{8}{3}$ and $\rho = 28$, respectively. The initial values of the two Lorenz systems are $\left[ {x_{01} ,y_{01} ,z_{01} \left] = \right[ - 0.823 ,3.423 , 0.178} \right]$ and $\left[ {x_{02} ,y_{02} ,z_{02} \left] = \right[ - 1.804 , - 0.631 , - 1.512} \right]$, respectively.

**Figure 3:**
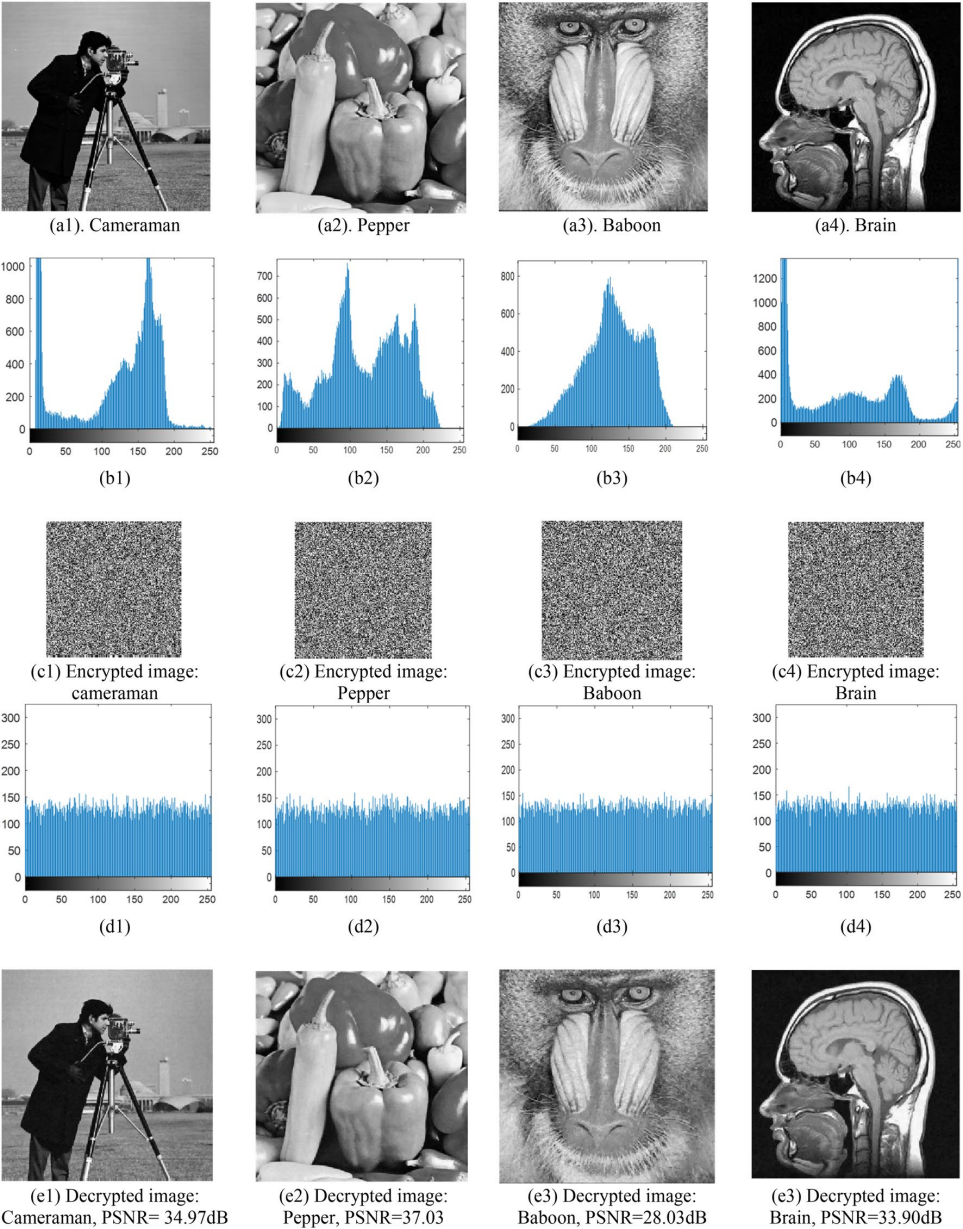
Experimental results: (**a1**–**a4**) original images; (**b1**–**b4**) the corresponding histograms of the original images; (**c1**–**c4**) the corresponding encrypted images of the original images; (**d1**–**d4**) the corresponding histograms of the encrypted images; (**e1**–**e4**) the corresponding decrypted images; Cameraman is used from the website https://homepages.cae.wisc.edu/~ece533/images/; Pepper and Baboon are used from the website http://sipi.usc.edu/database/; Brain scan is used from the website https://medicalxpress.com/news/2017-02-mri-brain-scans-adolescent-substance.html.

To evaluate the quality of the decrypted image, we use two measures of the peak signal-to-noise ratio PSNR and the mean square deviation MSE defined as follows:

9 $$\begin{gathered} MSE = \frac{1}{{n_{1} n_{2} }}\mathop \sum \limits_{i,j} \left( {{\mathbf{I}}\left( {i,j} \right) - {\hat{\mathbf{I}}}\left( {i,j} \right)} \right)^{2} \hfill \\ PSNR = 10\log_{10} \left( {\frac{{255^{2} }}{MSE}} \right) \hfill \\ \end{gathered}$$

where ${\mathbf{I}} \in R^{{n_{1} \times n_{2} }}$ and ${\hat{\mathbf{I}}} \in R^{{n_{1} \times n_{2} }}$ are the original image and the decrypted image, respectively. Figure 3 shows the results of image encryption for test images. In these examples, the histogram of the original image and encrypted image is also shown. These results indicate that all images have the same results visually and statistically. The decrypted images also have an acceptable quality.

### Compression performance

**Figure 4:**
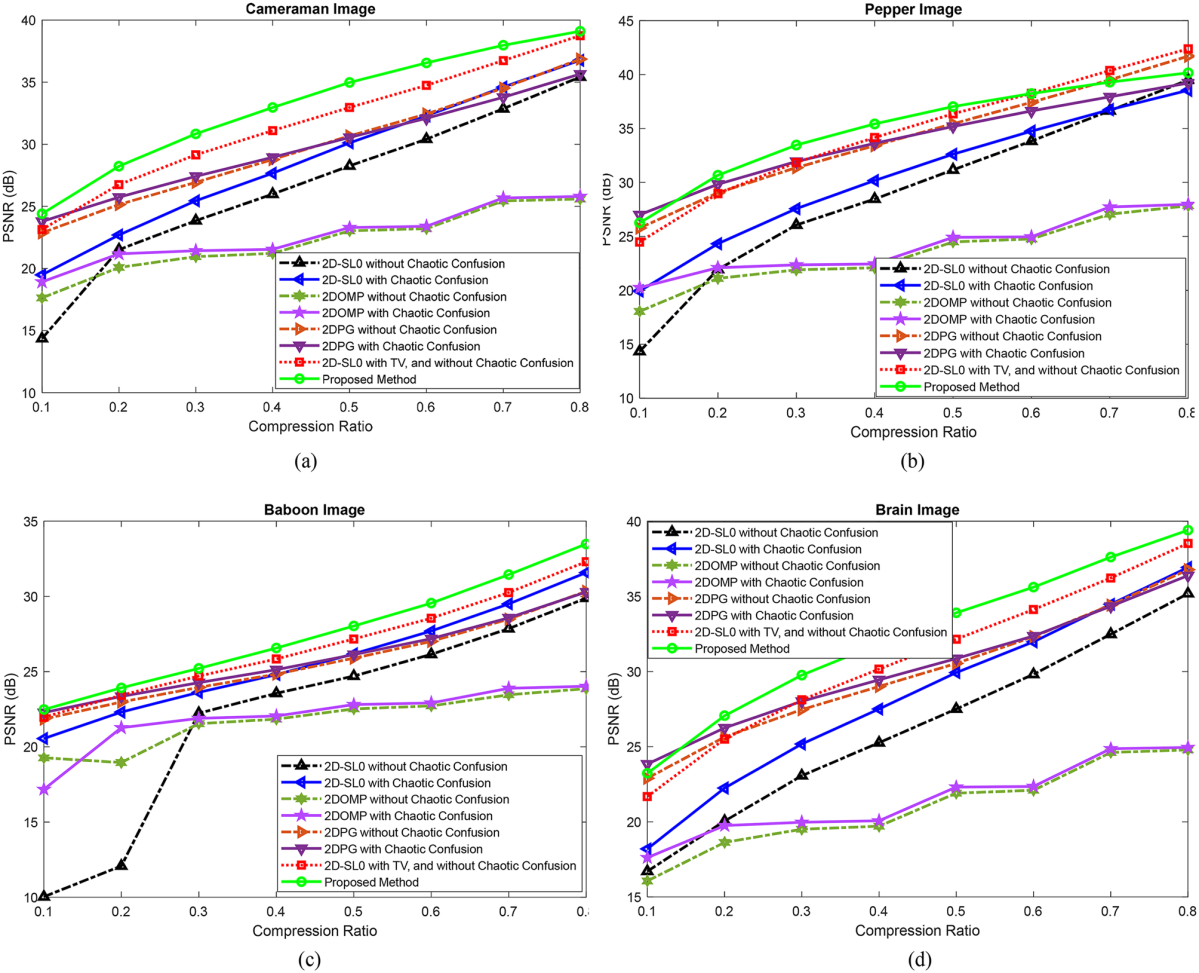
Performance of the sparse reconstruction approaches with considering the effects of TV and chaotic confusion in the encryption process: The PSNR values of the decrypted test images for different CRs.

In this section, the decrypted image quality is evaluated for different compression ratios based on the PSNR measure. In the proposed method, to obtain better performance of the image reconstruction, two techniques are used. The first one is based on the uniqueness analysis. The chaotic confusion is applied to the sparse representation of the original image. Also, TV is added to the cost function of sparse recovery. Here, the effect of these terms on the quality of the decrypted image is investigated. Figure 4 shows the PSNR value for different CRs. Here, the proposed approach is also compared with 2DOMP^57^ and 2DPG^58^ algorithms. We also evaluate the effect of chaotic confusion on these reconstruction approaches. The reconstruction results demonstrate that the technique of chaotic confusion improves the reconstruction performances of 2DSL0, 2DOMP, and 2DPG methods, especially for small compression ratios. These curves show that the image recovery quality has been enhanced with two applied techniques in 2D sparse representation. The proposed approach outperforms 2D-OMP and 2DPG reconstruction methods. Here, we also present the average computation time to compare the computational complexity of different reconstruction approaches in Table 1. Since the runtime depends on the programming skill and the programming languages, this criterion is not appropriate for a computation cost comparison.

**Table 1:** The computation time (s) for the different compression ratios.

Compression ratio	0.1	0.3	0.5	0.7
2DSL0	4.4	6.9	9.1	10.6
2DOMP	5.1	22.4	284.1	3753
2DPG	102.6	104.3	105.7	105.4
Proposed method	22.4	27.1	30.3	33.6

Table 2 presents the performance of different schemes based on compressed sensing in comparison with the proposed approach. It can be seen that the proposed encryption approach has a better performance for the same CR. Table 3 shows the performance of the decrypted images for different CRs visually. The qualities are entirely acceptable.

**Table 2:** PSNR (dB) values of the encrypted images with different approaches for the different compression ratios.

Image	Compression ratio	Proposed method	Ref.^45^	Ref.^30^	Ref.^40^	Ref.^26^
Cameraman	0.25	29.58	–	–	25.23	24.24
	0.50	34.97	$\approx$ 25.08	$\approx$ 25.64	29.43	30.66
	0.75	38.61	26.81	27.45	28.93	31.08
Pepper	0.50	37.03	$\approx$ 27.33	$\approx$ 27.94	–	32.09
	0.75	39.70	28.67	29.19	–	–

**Table 3:**
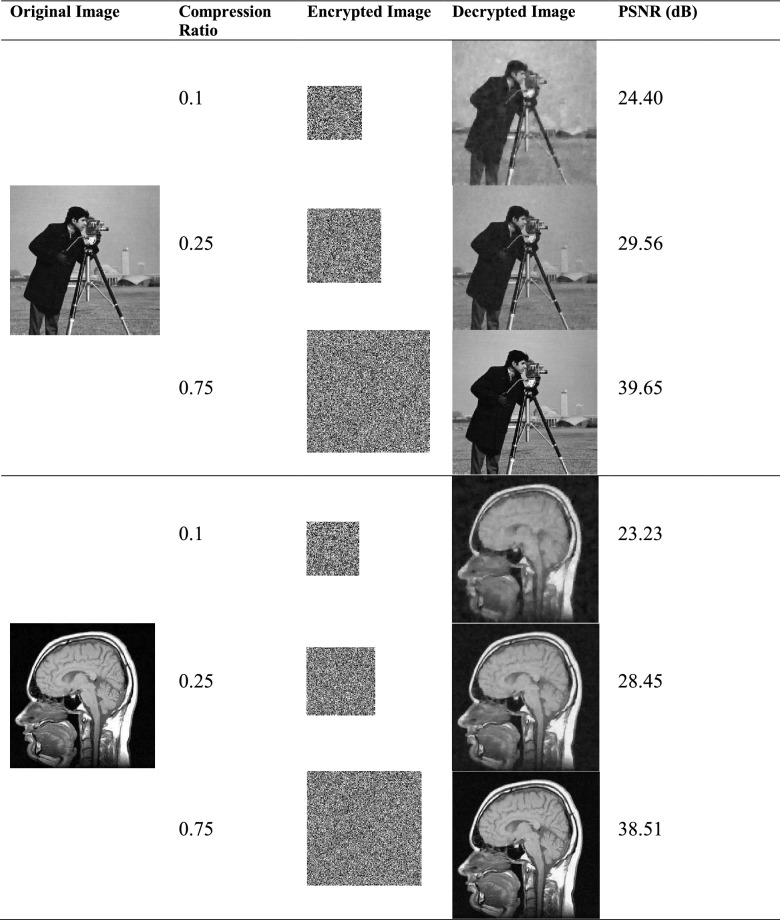
The results of different compression ratios.

### Performance evaluation of the proposed measurement matrix

In this section, we evaluate the effectiveness of the proposed measurement matrix. This matrix is a partial orthogonal matrix generated by the chaotic sequence and SVD. Here, we compare the proposed measurement matrix with the partial Hadamard matrix and the Gaussian random matrix. Partial Hadamard matrix, a famous measurement matrix, has been used in encryption schemes^22,35,38,39,45^. Table 4 shows the effectiveness of these measurement matrices in the proposed encryption process for the different CRs. It can be observed that the proposed measurement matrix has better performance than the Gaussian matrix in this application. Also, this presents similar PSNR values compared with that of the partial Hadamard matrix. Therefore, the proposed measurement matrix achieves good performance and effectiveness in compressed sensing and increases image encryption security.

**Table 4:** PSNR (dB) values of the encrypted images with different measurement matrices.

Image	CR	Proposed measurement matrix	Gaussian random matrix	Partial hadamard matrix
Cameraman	0.1	24.40	23.50	24.59
	0.3	30.83	28.18	30.71
	0.5	34.97	29.66	35.66
Pepper	0.1	26.25	25.19	25.97
	0.3	33.46	29.39	32.79
	0.5	37.03	30.46	37.80
Baboon	0.1	22.48	22.38	22.03
	0.3	25.21	24.25	24.87
	0.5	28.03	25.47	27.86
Brain	0.1	23.23	22.19	22.99
	0.3	29.75	28.80	29.21
	0.5	33.90	29.34	34.02

### Security performance analyses

In this section, the security performance of the proposed approach is analyzed by two measures. The initial value of the chaotic system is keystream used to encrypt different images. To increase the image encryption security level in different attacks, it is appropriate to generate a keystream related to the original image. To obtain this purpose, many approaches can be proposed. For example, inspired by Ref.^30^, we can use the SHA-256 hash value of the original image to generate the chaotic system's initial value. Hence, when different images are encrypted, the corresponding measurement matrices and chaotic confusion operators are different. This can ensure that the proposed approach can resist various attacks, such as the chosen-plaintext attack, which is the strongest attack.

#### Key sensitivity

**Figure 5:**
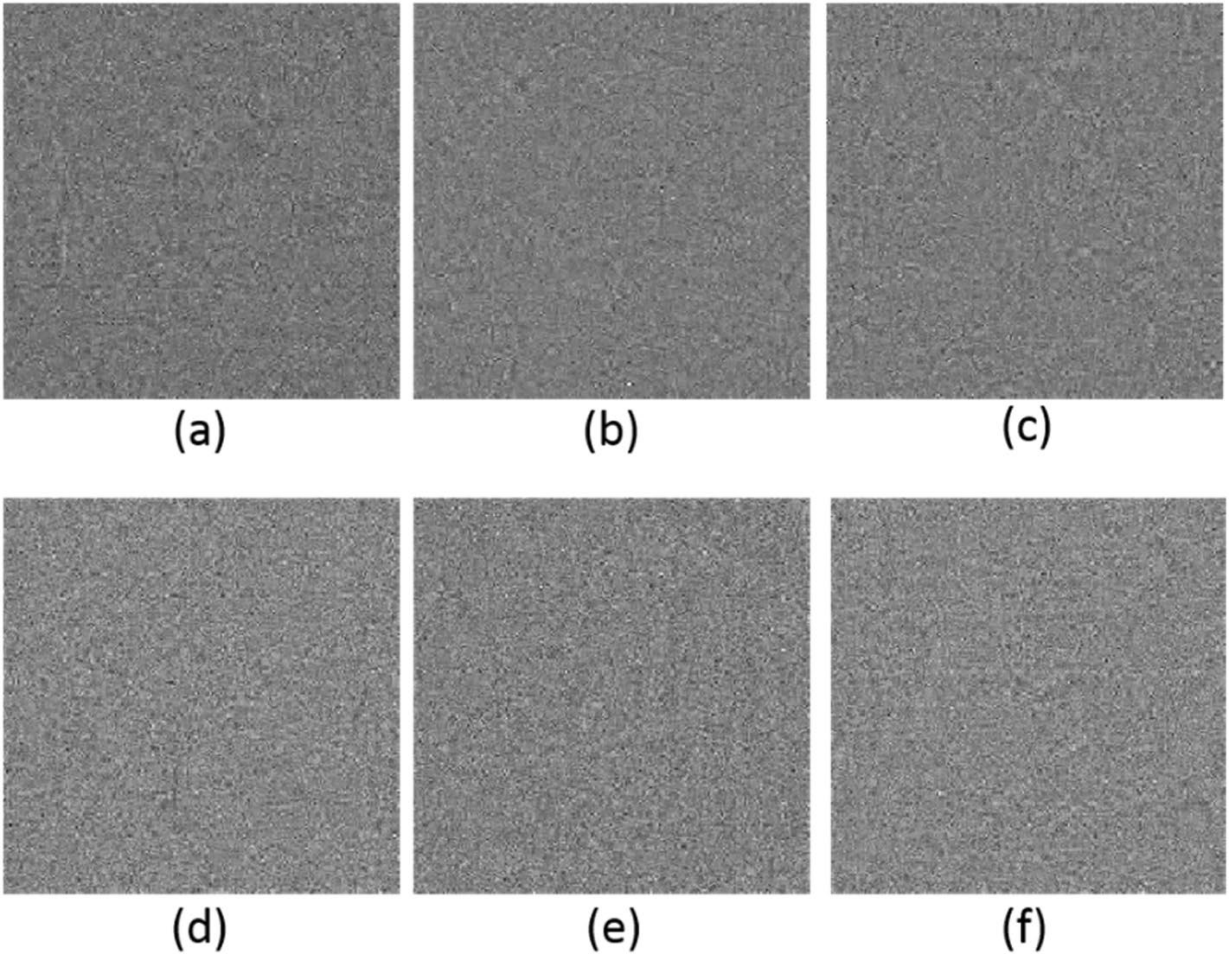
Decrypted Cameraman image with incorrect keys: (**a**) decrypted image with $x_{01} + 10^{ - 15}$; (**b**) decrypted image with $y_{01} + 10^{ - 15}$; (**c**) decrypted image with $z_{01} + 10^{ - 15}$; (**d**) decrypted image with $x_{02} + 10^{ - 15}$; (**e**) decrypted image with $y_{02} + 10^{ - 15}$; (**f**) decrypted image with $z_{02} + 10^{ - 15}$.

**Figure 6:**
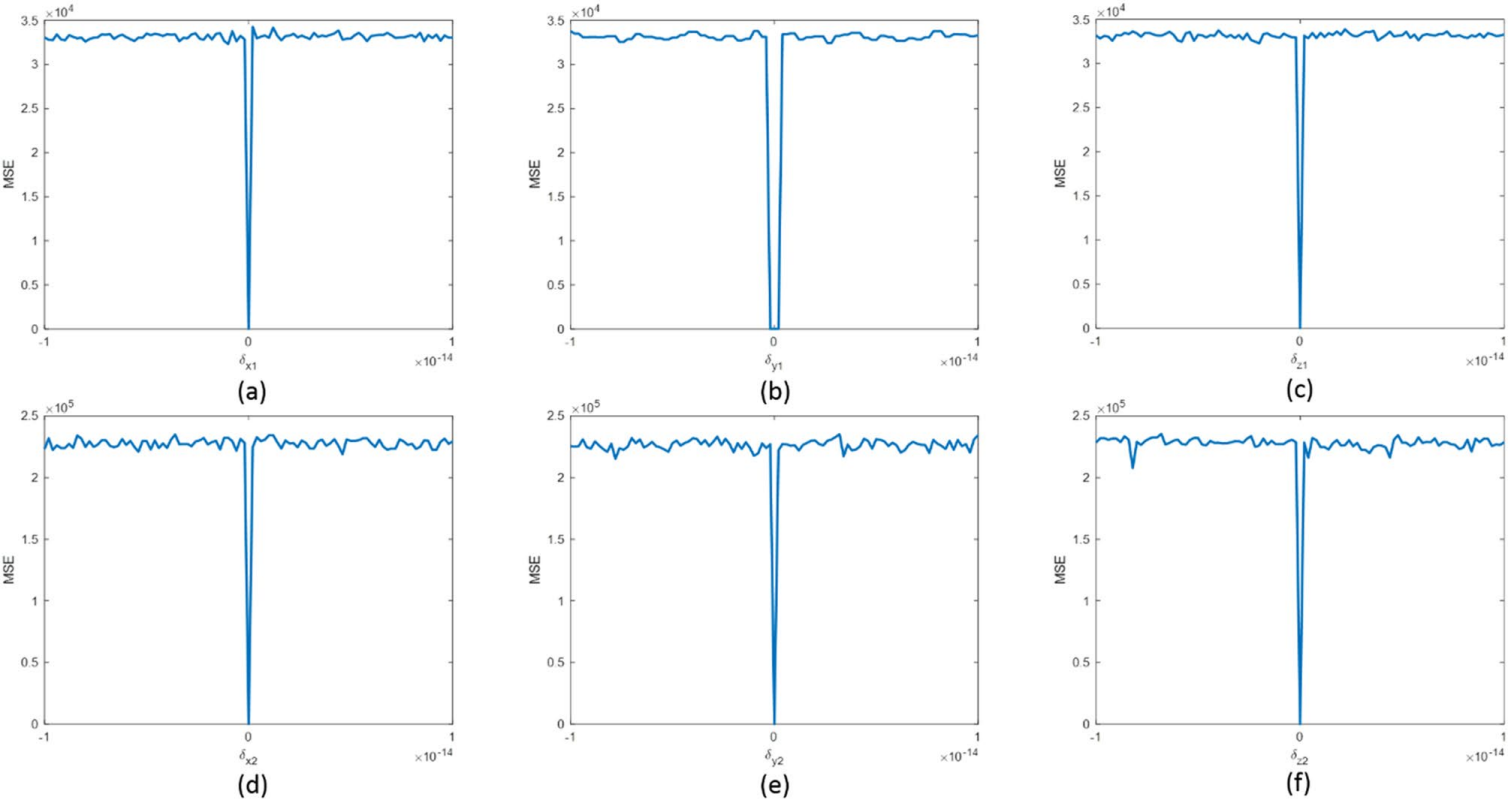
MSE curves concerning the variation of each key: (a) $x_{01} + \delta_{x1}$; (**b**) $y_{01} + \delta_{y1}$; (**c**) $z_{01} + \delta_{z1}$; (**d**) $x_{02} + \delta_{x2}$; (**e**) $y_{02} + \delta_{y2}$; (**f**) $z_{02} + \delta_{z2}$.

The key sensitivity analysis shows the effect of a tiny difference between decryption and encryption keys on the quality of the decrypted image. In a secure cryptosystem, a tiny variation in the secret key causes to obtain a decrypted image, which is entirely different from the original image. In the proposed approach, the sensitivity of the chaotic system to the initial conditions results in a secure scheme to the keys $\left[ {x_{01} ,y_{01} ,z_{01} , x_{02} ,y_{02} ,z_{02} } \right]$. Figure 5 shows the decrypted Cameraman images for different wrong keys. In each key set, only one key has a tiny variation of $10^{ - 15}$, and the other keys are correct. It is obvious that the decrypted image has been significantly distorted. Figure 6 illustrates the key sensitivity based on the MSE measure for the variation of each key. It can be seen that the minimum of MSE is obtained when the keys are correct. Put it in a nutshell, the proposed method is highly sensitive to the secret keys.

#### Key space

An essential requirement of a secure cryptosystem is the key space. The larger key space makes the brute force attack infeasible. According to the research^61^, the key space should be larger than $2^{100}$. The precision of the double-precision number is about $10^{ - 15}$. Therefore, the total key space of the proposed encryption approach is $\left( {10^{15} } \right)^{6} = 10^{90} \approx 2^{300}$ which is large enough. This key space prevents the exhaustive searching. Table. 5 compares the key space of different approaches.

**Table 5:** Key space of the proposed method and other approaches.

Algorithm	Proposed method	Ref.^45^	Ref.^30^	Ref.^40^	Ref.^26^
Key space	$2^{300}$	$2^{276}$	$2^{300}$	$2^{232}$	$2^{398}$

### Statistical analyses

In this subsection, the effectiveness of the proposed encryption scheme is investigated from the statistical viewpoint.

#### Histogram

A histogram is a measure to evaluate the performance of the encryption approach. An appropriate histogram of a secure cryptosystem is a uniform distribution that makes the encryption process resistant to the statistical attack. Figure 3 shows the histograms of original and encrypted images. It can be seen that the proposed method changes approximately the histogram of all test images to the uniform. These results demonstrate the effectiveness of the proposed approach.

#### Entropy

Information entropy is another measure to calculate the randomness of the encrypted image. The larger value of entropy means the better performance of encrypted images. For a discrete variable, the Shannon entropy is maximized for the uniform distribution. The maximum value is equal to $\log_{2} M$ where $M$ is the number of discrete variable states. In the encryption scheme, this value is equal to 8 by considering 8 bits for each pixel. Table 6 shows the entropy of the original and encrypted images for $CR = 0.5$. The results describe that the entropy after encryption is approximately equal to the ideal value.

**Table 6:** The entropy of the original and encrypted images.

	Original image	Encrypted image
Cameraman	7.0097	7.9945
Pepper	7.7721	7.9949
Baboon	7.5703	7.9941
Brain	6.9868	7.9939

#### Correlation

**Figure 7:**
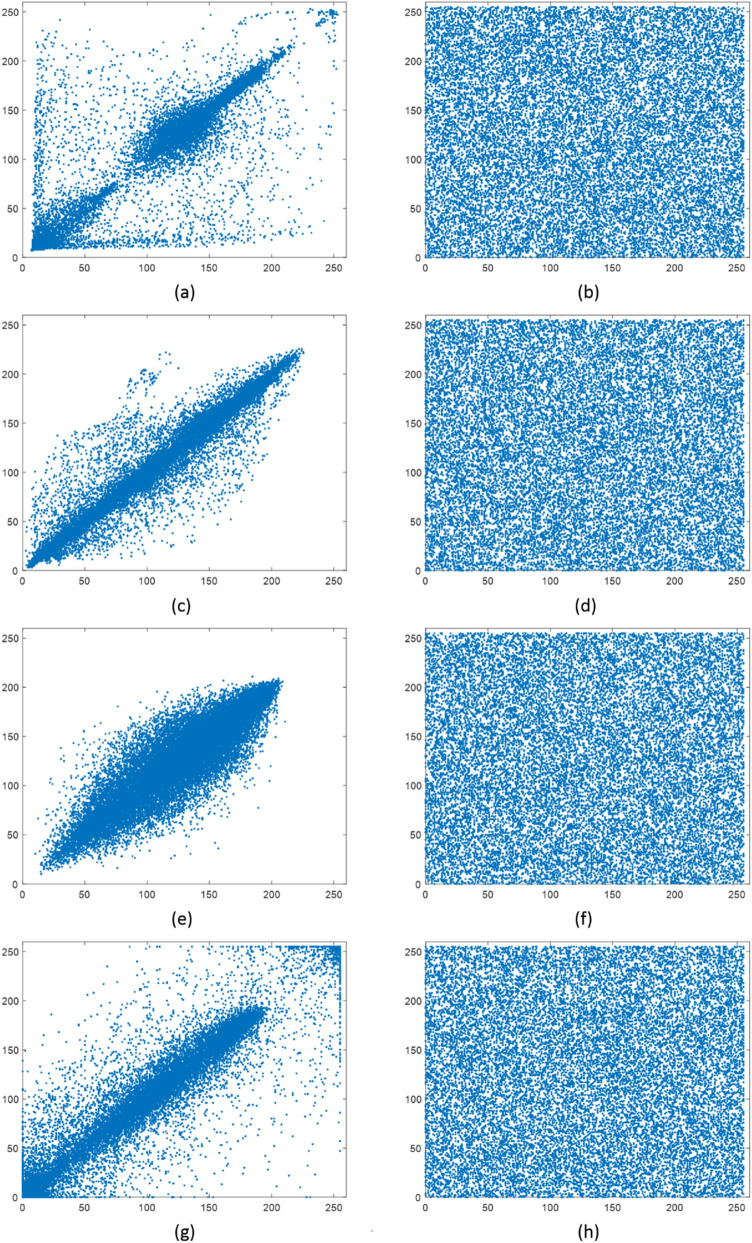
The correlation distribution of two adjacent pixels in the horizontal direction for the original and encrypted images. (**a**) Original “Cameraman”; (**b**) encrypted “Cameraman”; (**c**) original “Pepper”; (**d**) encrypted “Pepper”; (**e**) original “Baboon”; (**f**) encrypted “Baboon”; (**g**) original “Brain”; (**h**) encrypted “Brain”.

Two previous measures investigate the uniformity of the distribution while they do not calculate the correlation among pixels. For a natural image, there is a high correlation between adjacent pixels in all directions. The minimum correlation among pixels is appropriate for secure image encryption. Figure 7 shows the joint distribution of adjacent pixels in the horizontal direction for the original and encrypted images. These distributions show that the correlation between pixels of the encrypted images is reduced effectively. A measure to quantify the relation between the adjacent pixels of the image is the correlation coefficient (CC). Table 7 presents the CC values of different schemes in three directions for comparison. The results show that the proposed encryption method reduces the correlation between pixels, and has better performance.

**Table 7:** Correlation coefficients of adjacent pixels.

Image	Algorithms	Horizantal	Vertical	Diagonal
Cameraman	Original image	0.9592	0.9337	0.9079
	Proposed method	0.0014	− 0.0044	− 0.0031
	Ref.^45^	–	–	–
	Ref.^36^	− 0.0044	− 0.0054	0.0025
	Ref.^26^	0.0040	− 0.0027	− 0.0084
Pepper	Original image	0.9714	0.9644	0.9388
	Proposed method	0.0033	− 0.0016	− 0.000058
	Ref.^45^	− 0.0005	− 0.0062	0.0036
	Ref.^36^	0.0074	0.0035	0.0041
	Ref.^26^	− 0.0117	0.0039	− 0.0012

### Roustness analysis

#### Noise attack

**Figure 8:**
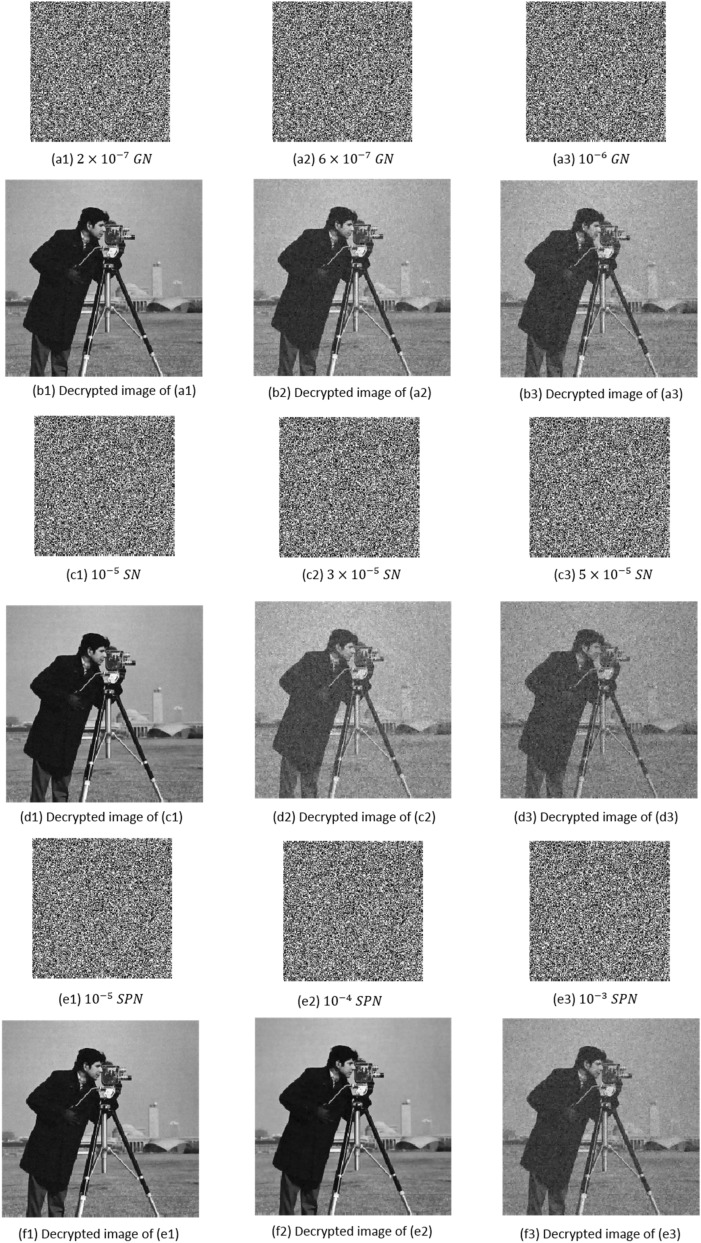
Encrypted images and decrypted images in the presence of GN, SN, and SPN with different levels of noise.

**Figure 9:**
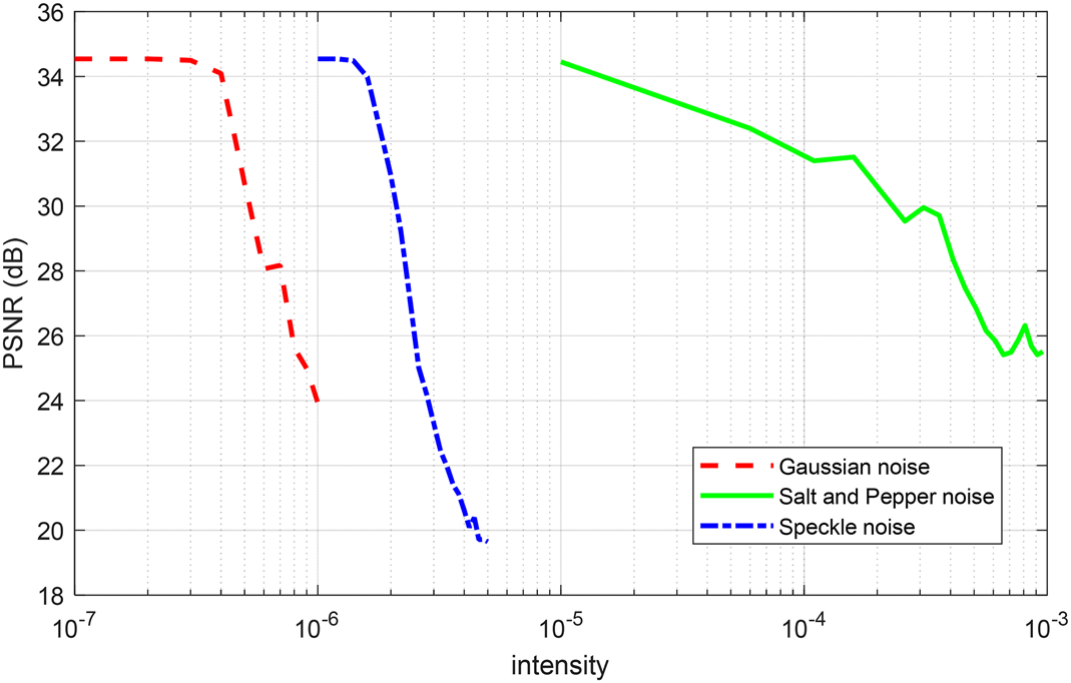
PSNR between decrypted images and original images with different noises.

Images are usually affected by different noise models in the transmission process. Here, we evaluate the robustness of the proposed approach in the presence of different noise attacks such as Gaussian noise (GN), speckle noise (SN), and salt and pepper noise (SPN). In this simulation, the Cameraman image is used as a test image, and $CR = 0.5$. Figure 8 presents decrypted images in the presence of noise attacks with different intensities. The recovery performance of the proposed encryption approach is shown in Fig. 9 for different noise intensities. We can conclude that: (1) Gaussian noise has the largest effect on the recovery performance, (2) there is a certain robustness to the SN attack, (3) the proposed scheme has the strongest robustness to the SPN attack. These results show that the proposed encryption method has a good ability to recover the original image in the presence of the noise attack.

#### Cropping attack

**Figure 10:**
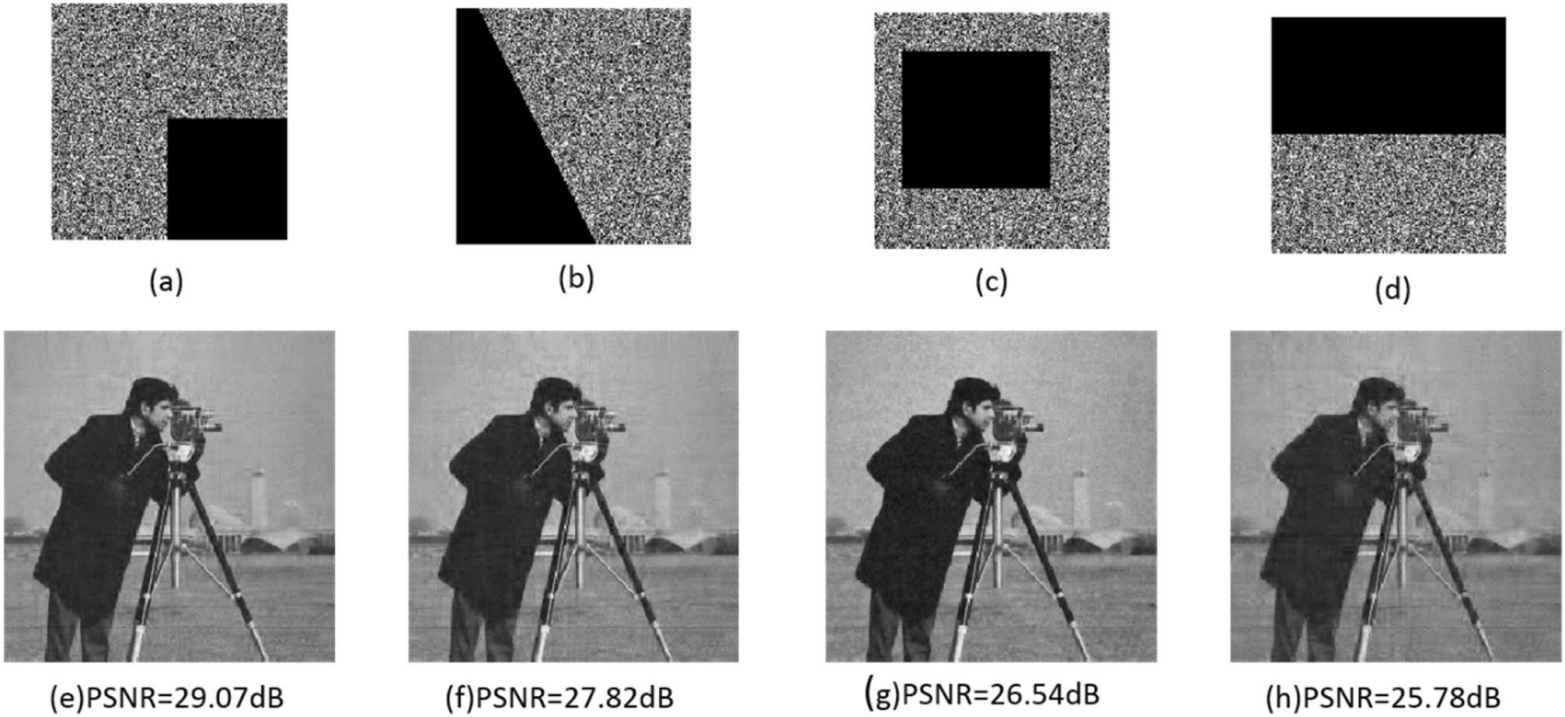
Result of cropping attacks: (**a**–**d**) encrypted images with different cropping attacks; (**e**–**h**) the decrypted images from (**a**–**d**), respectively.

An essential challenge in image transmission is the cropping attack in which some pixels of the encrypted image are loosed. Since the proposed image encryption is based on compressed sensing, our approach is robust against data loss and can effectively recover the original image. The proposed approach's performance is evaluated for different cropping masks, and results are shown in Fig. 10. The decrypted images and their PSNR demonstrate the robustness of the proposed method in the presence of a cropping attack.

## Conclusion

In this paper, an image cryptosystem was proposed based on two-dimensional sparse recovery to simultaneously achieve encryption and compression. Based on the compressed sensing theory, the original image was compressed via two measurement matrices. To improve the performance of image recovery, a chaotic confusion was added before compression. Also, the total variation was used in the sparse recovery problem based smoothed $\ell_{0}$ norm. The security level was increased by chaotic scrambling and XOR operations. Statistical analyses have shown that the proposed cryptosystem has ample key space, high sensitivity to the tiny variation in the key, maximum entropy, uniform distribution of the encrypted image, and week correlation between adjacent pixels. These features have demonstrated the robustness of the proposed approach against the statistical analysis and brute-force attack. The simulation results have illustrated that the proposed cryptosystem has high security and excellent compression performance. The proposed approach was also adequately robust against the noise and cropping attacks because of considering the chaotic confusion and the total variation in the two-dimensional sparse recovery. Future work will focus on improving the compression ratio performance based on the dictionary learning to obtain the sparse representation.
